# Replacing chemical fertilizers with organic fertilizers at equivalent nitrogen levels enhances soil nitrogen transformation rates in Northwest China’s farmlands, consequently impacting crop yields

**DOI:** 10.3389/fpls.2026.1772980

**Published:** 2026-03-03

**Authors:** Xiaohua Shi, Yuanyuan Zhang, Junmei Liang, Liguo Jia, Yonglin Qin, Yang Chen, Jing Yu, Kun Liu, Lan Wu, Bofeng Zhou, Hongli Zhen, Mingshou Fan

**Affiliations:** 1 Inner Mongolia Agricultural University, Hohhot, China; 2 Inner Mongolia Academy of Agricultural & Animal Husbandry Sciences, Hohhot, China

**Keywords:** arid and semi-arid region, crop yield, organic fertilizer substitution, soil enzyme activity, soil nitrogen forms

## Abstract

The effectiveness of substituting organic fertilizers for chemical fertilizers in enhancing crop yield and soil quality remains uncertain. This study conducted a two-year field experiment to investigate the effects of replacing chemical fertilizer nitrogen with organic fertilizer nitrogen at varying levels (0%, 30%, 60%, 100%) on soil nitrogen transformation and crop yield in potato and wheat cultivation. We measured soil nitrogen pools and key nitrogen-transforming enzyme activities to evaluate how various fertilization treatments affect the soil nitrogen cycling process. Results showed that replacing 60% of chemical fertilizer nitrogen with organic fertilizer (T2) significantly increased soil nitrate nitrogen content during the potato tuber expansion and wheat jointing stages. It also maintained high mineral nitrogen levels in the later growth period. Additionally, the T2 treatment significantly boosted soil urease (SU) activity by 14 - 38% and Soil alkaline protease (ALPT) activity by 9 - 22%, optimizing nitrogen transport to potato tubers and wheat grains. Compared to full chemical fertilizer treatment, T2 increased potato yield to 47.13 t·ha^-^¹ and wheat yield to 6.06 t·ha^-^¹, marking increases of 18.8% and 22.8%, respectively, and improved nitrogen use efficiency by 73.4 - 76.1%. This study demonstrates that substituting 60% of chemical fertilizer nitrogen with organic fertilizer effectively meets crop needs through a “quick-release and slow-release” coordinated nitrogen supply mechanism. It enhances nutrient release by boosting soil enzyme activities during critical fertilizer-demanding periods, offering a viable solution for reducing fertilizer use and increasing efficiency in arid and cool regions.

## 1 Introduction

Farmland soils in Northwest China are infertile, and the ecosystem is fragile. Despite these challenges, the region is a major global potato producer. Reliance on chemical fertilizers to boost yields over the long term has worsened issues like soil desertification, salinization, and fertility decline, significantly hindering sustainable agricultural development (Ankita et al., 2024^;^Ju et al., 2024). Therefore, implementing a strategy to “reduce chemical fertilizer use while increasing efficiency” is urgently needed to balance high yields with environmental protection, facilitating the region’s green agricultural transformation (Rongxiu et al., 2025; Chenxiao et al., 2023). In Northwest China, cropland fertilization has traditionally relied on mineral fertilizers, particularly urea-based nitrogen, to offset low soil fertility and maintain yields in arid and semi-arid environments. Recently, fertilization strategies have shifted from “high-input yield maintenance” to “input reduction with efficiency improvement.” This change is driven by soil-testing-based formula fertilization, integrated water-fertilizer management, and the growing feasibility of manure recycling. Despite these advancements, the region still encounters several challenges, including nutrient imbalance, low nitrogen use efficiency, and a mismatch between rapid mineral nitrogen supply and crop nitrogen demand. Additionally, soil degradation risks, such as salinization, alkalinization, and structural decline, persist. The effectiveness of organic fertilizer substitution is often limited by low temperatures and water scarcity, which suppress microbial activity and delay organic nitrogen mineralization. This delay increases uncertainty in early-stage nitrogen supply and yield stability.

In Inner Mongolia, organic fertilizer resources are abundant. Utilizing these resources and replacing chemical fertilizers with organic alternatives can increase soil organic matter, promote nutrient slow-release, and improve crop quality. This approach also addresses the conflict between agricultural production and environmental sustainability (Mateusz et al., 2024; Gholamreza et al., 2024). However, most research has focused on the macro-effects of partially substituting organic fertilizer nitrogen on soil fertility and crop yield. A more comprehensive understanding of the nitrogen transformation process and its enzymatic regulation in specific regions and crop systems is necessary to grasp the soil-crop system’s response to coordinated organic-inorganic nitrogen management (Muhammad et al., 2022^;^Qinghua and Wenliang, 2024; Michail L. et al., 2023; Paul and Urs, 2021). The agro-pastoral ecotone at the northern foot of the Yinshan Mountains in Inner Mongolia is a typical arid and semi-arid area. While preliminary evidence suggests that replacing chemical fertilizers with organic ones improves soil structure and fertility, there is a lack of reliable field-experiment-based evidence on its effects on soil nitrogen hydrolase activity, nitrogen transformation processes, and nitrogen utilization efficiency (Chenxiao et al., 2023; Fengwei et al., 2024; Yibing et al., 2024; Jia et al., 2023; Tufail et al., 2023). Understanding the relationships among these factors is crucial for comprehending the microbial and biochemical mechanisms of nitrogen cycling driven by organic fertilizers under arid and cool conditions (Ubaldi et al., 2023; Kerner et al., 2023). This knowledge is essential for scientifically evaluating the nitrogen-substitution effect of organic fertilizers and optimizing regional nutrient management strategies. Conducting relevant field experiments in this region holds significant scientific value. It promotes the efficient and precise application of organic fertilizers and supports the coordinated development of effective agricultural nitrogen utilization and environmental protection.

A two-year field experiment was conducted in the chestnut soil area of Ulanqab City, Inner Mongolia, to evaluate the feasibility of reducing chemical fertilizers without decreasing yields. The study involved substituting chemical fertilizer nitrogen with organic fertilizer nitrogen at gradients of 0%, 30%, 60%, and 100%, while maintaining equal nitrogen levels. We hypothesized that substituting chemical fertilizer nitrogen with organic fertilizer nitrogen would better align the soil’s nitrogen release peak with the crops’ maximum nitrogen demand period. This alignment could enhance nitrogen use efficiency and boost crop yields. To understand the biological mechanisms behind how partial substitution affects the soil nitrogen pool and crop productivity, we analyzed the synergistic effects of different substitution ratios. Our focus was on the transformation of soil nitrogen forms, the activities of key nitrogen-transforming enzymes, and nitrogen utilization and yield formation during potato and wheat cultivation. This study provides a theoretical foundation and technical support for developing a green fertilization technology system tailored for potatoes and wheat in arid and semi-arid regions. It also aims to improve the resource utilization efficiency of regional agricultural waste and support the achievement of the “dual carbon” goal.

## 2 Materials and methods

### 2.1 Experimental site

In this experiment, potatoes were planted from May to September 2022, while wheat was planted from April to August 2023. The experimental field was situated in Kebuer, Chayouzhong Banner, Ulanqab City, Inner Mongolia (112°36′10.41″E, 41°18′2.31″N), at an altitude of 1780 meters. The region experiences an average annual precipitation of 304 mm and an average temperature of 1.3 °C. Annual evaporation ranges from 210 to 420 mm, and the frost-free period lasts approximately 100 days. The soil type in the experimental field is Castanozems. The operational depth of a roll-over plow ranges from 35 to 40 cm, thus defining the 0–40 cm layer as the cultivated layer. Detailed physical and chemical properties of the soil in the 0–40 cm plow layer before sowing are provided in **Table 1**.

**Table 1:** Basic physical and chemical properties of the soil in the experimental area.

pH	Organic matter (%)	Total nitrogen (g/kg)	Available phosphorus (mg/kg)	Available potassium (mg/kg)	Bulk density (mg/kg)
8.30	2.02	1.26	8.91	113.7	1.35

### 2.2 Experimental design

The potato variety tested was “Kexin No. 1, “ developed by Inner Mongolia Xufeng Agricultural Technology Co., Ltd. The wheat variety was “Yongliang No. 4, “ produced by Kehe Seed Industry Co., Ltd. in Bayannur City, Inner Mongolia. The organic fertilizer used in the study was manufactured by Baotou Madison Ecological Plant Technology Co., Ltd., with its nutrient content detailed in **Table 2**-2. The chemical fertilizers tested included urea (N ≥ 46%), triple superphosphate (P_2_O_5_ ≥ 46%), and potassium sulfate (K_2_O ≥ 51%).

**Table 2:** Nutrient content of organic fertilizers (%).

Organic matter	N	P_2_O_5_	K_2_O
46	2.14	1.24	2.18

The experimental field employs drip irrigation. Potatoes are planted with a ridge spacing of 90 cm and a plant spacing of 24 cm, resulting in a planting density of 46, 000 plants per hectare. Wheat is sown at a rate of 300 kg·ha^-1^, with a sowing density of 7×10^6^ plants·ha^-1^. In 2022, potatoes were sown on May 3 and harvested on September 14. In 2023, wheat was sown on April 19 and harvested on August 14.

The experiment utilized a randomized block design with five treatments: no nitrogen fertilizer as the control (CK), full chemical fertilizer nitrogen (T0), 30% replacement of chemical fertilizer nitrogen with organic fertilizer (T1), 60% replacement (T2), and 100% replacement (T3). Among the five treatments, all except the CK treatment reflect common fertilization strategies used in local agriculture and nutrient management policies. The T0 treatment aligns with the conventional fertilization strategy in the region. The 30% and 60% partial substitutions are widely accepted by farmers and extension projects in Northwest China, whereas the 100% full substitution is typical in traditional agriculture. **Table 3** details the fertilization amounts for each treatment. All fertilizers were applied as basal fertilizers, with consistent management measures across treatments. Each treatment was replicated four times. The plot area measured 9 m × 10 m, with a 1.8 m isolation row between plots.

**Table 3:** Experimental design.

Crop	Treatment	Organic fertilizer (kg·ha^-1^)	Fertilizer dosage (kg·ha^-1^)			Replacement ratio
			N	P_2_O_5_	K_2_O	N
Potato	CK	0	0	210	315	0
	T0	0	300	210	315	0
	T1	4200	210	210	315	30
	T2	8400	120	210	315	60
	T3	14000	0	210	315	100
Wheat	CK	0	0	90	150	0
	T0	0	180	90	150	0
	T1	2500	126	90	150	30
	T2	5000	73	90	150	60
	T3	8400	0	90	150	100

The substitution ratio should be rounded to the tens place.

### 2.3 Sample collection and analysis

During the experiment, soil samples from the 0–40 cm plow layer were collected at each growth stage. For each sampling, five points were selected in an “S” pattern within each treatment plot. These samples were analyzed for soil moisture content, total nitrogen (TN), nitrate nitrogen (NO_3_^-^ - N), ammonium nitrogen (NH_4_^+^ - N), and the activities of soil urease (SU) and soil alkaline protease (ALPT). During the potato-growing season, plant samples were collected at various stages: seedling, tuber formation, tuber expansion, starch accumulation, and maturity. Five plant samples were randomly selected from each plot. In the wheat-growing season, samples were taken at the seedling, tillering, booting, filling, and maturity stages. Three 1m² samples were randomly selected from each plot to measure nitrogen absorption and transport in each plant organ, as well as the yield of economic organs and the commodity rate.

The total nitrogen content in the plants was measured using the Dumas combustion method with a Primacs SN100 Dumas nitrogen analyzer (Skalar, Holland).

Soil NO_3_^-^-N and NH_4_^+^-N concentrations were measured with a San++ Compact V2 continuous flow analyzer (Skalar, Holland).

Activity tests for SU and ALPT were conducted using detection kits from Suzhou Keming Biotechnology Co., Ltd., China.

### 2.4 Statistical analysis

Regularly measure the mineralizable nitrogen content in the soil to verify the sustained-release properties of organic fertilizers.

Nitrogen Use Efficiency (NUE, %) is calculated using the formula: NUE = (Crop nitrogen uptake in nitrogen application treatments - Crop nitrogen uptake in the CK)/Nitrogen application.

Nitrogen accumulation is calculated by multiplying the dry matter weight per unit area at a specific growth stage (such as straw, grain, stems/leaves, or tubers) by the nitrogen content.

Data were organized using Microsoft Excel 2021. Bar and stacked bar charts were plotted with GraphPad Prism, while SPSS 26.0 software facilitated one-way analysis of variance. The Pearson correlation method analyzed correlations between various soil nitrogen forms and environmental factors, with significance levels set at P < 0.05 and P < 0.01. Canoco5 software conducted redundancy analysis to explore relationships between soil nitrogen content and environmental factors. IBM SPSS Amos 23.0 software constructed a structural equation model (SEM) to evaluate the direct and indirect impacts of soil nitrogen forms, nitrogen-transforming enzyme activities, and crop yield on various indicators.

## 3 Results and analysis

### 3.1 Potato and wheat yields and yield components

**Table 4** illustrates that potato yield initially increased and then decreased as the organic fertilizer substitution ratio rose. The T2 treatment, where 60% of chemical fertilizer nitrogen was replaced with organic fertilizer, produced a significantly higher tuber yield of 47.13 t·ha^-^¹ compared to other treatments (P < 0.05). This yield was 18.8% greater than that of T0, which used full chemical fertilizer. Conversely, the full substitution treatment (T3) resulted in a 9.3% yield decrease compared to T2 (P < 0.05). The yield increase was primarily due to a rise in both tuber number and individual tuber weight. Regarding commercial rate, T2 achieved 78.5%, significantly surpassing the control (CK) at 69.8% (P < 0.05), suggesting that appropriate organic fertilizer substitution can enhance tuber quality.

**Table 4:** Potato yield and its components.

Treatment	Final tuber number (piece/plant)	Fresh tuber yield (g/plant)	Commodity Rate (%)	Yield (t·ha^−1^)
CK	4.89 ± 0.42 c	0.79 ± 0.00 c	69.80 ± 1.35 b	33.12 ± 0.16 c
T0	6.44 ± 0.42 b	1.01 ± 0.06 b	76.65 ± 1.32 a	42.24 ± 2.51 b
T1	6.89 ± 0.57 b	0.99 ± 0.05 b	74.39 ± 0.69 ab	41.31 ± 2.20 b
T2	8.22 ± 0.16 a	1.13 ± 0.04 a	78.50 ± 3.53 a	47.13 ± 1.79 a
T3	7.22 ± 0.16 b	1.04 ± 0.01 ab	77.53 ± 3.49 a	43.39 ± 0.28 ab

The wheat yield performance was comparable to that of potatoes (**Table 5**). Under the T2 treatment, where 60% of chemical fertilizer nitrogen was replaced by organic fertilizer, the yield reached 6058 kg·ha^-^¹. This was a 22.8% increase over the T0 treatment, which used full chemical fertilizer (P < 0.05). The improvement was primarily attributed to a significant rise in the number of effective spikes and the 1000-grain weight. However, the yield under the full-replacement treatment (T3) decreased by 20.7% compared to the T2 treatment (P < 0.05). This suggests that excessive replacement may hinder grain filling due to delayed nitrogen supply.

**Table 5:** Wheat yield and its components.

Treatment	Effective spike number (×10^4^·ha^−1^)	Grain number per spike (g/plant)	1000- grain weight (g)	Yield (t·ha^−1^)
CK	374.3 ± 27.0 c	27.4 ± 2.8 b	36.7 ± 1.7 c	3109 ± 325 c
T0	443.0 ± 21.2 ab	34.4 ± 2.3 a	39.5 ± 2.0 bc	4932 ± 274 b
T1	448.9 ± 17.1 ab	36.9 ± 1.4 a	41.1 ± 1.4 ab	5384 ± 277 b
T2	479.6 ± 22.4 a	37.4 ± 1.5 a	43.9 ± 1.2 a	6058 ± 301 a
T3	421.8 ± 16.4 b	35.2 ± 2.1 a	38.6 ± 0.7 bc	4806 ± 261 b

### 3.2 Soil nitrogen transformation characteristics

**Figure 1:**
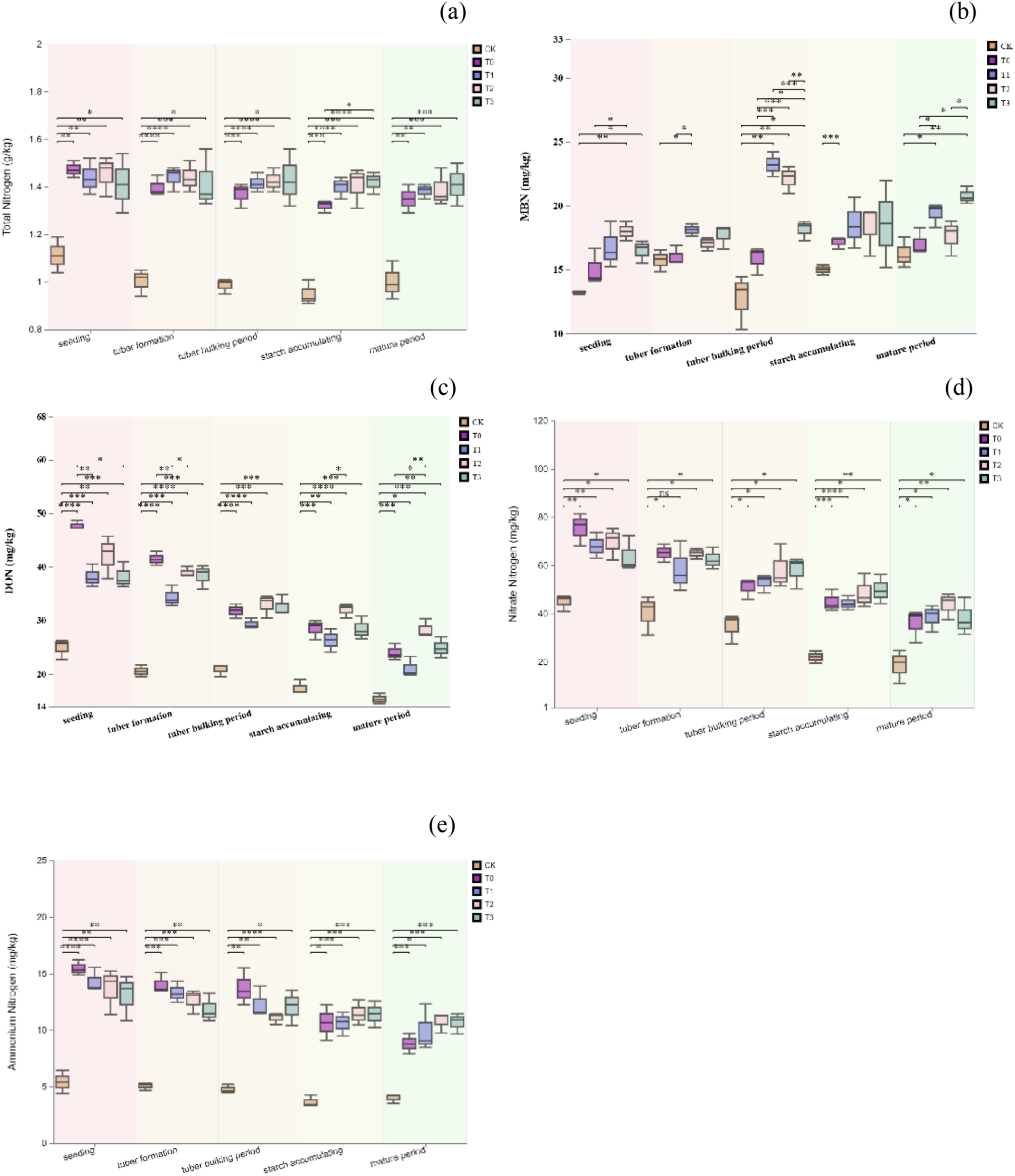
Total soil nitrogen **(a)**, Soil microbial biomass nitrogen **(b)**, Soil soluble organic nitrogen **(c)**, Soil nitrate nitrogen **(d)**, Soil ammonium nitrogen **(e)** during the potato season. A single asterisk (*) denotes a p-value less than 0.05, indicating statistical significance. Two asterisks (**) signify a p-value below 0.01, suggesting a higher level of significance. Three asterisks (***) represent a p-value under 0.005, indicating even greater significance. Four asterisks (****) denote a p-value less than 0.001, reflecting the highest level of statistical significance.

**Figure 1** illustrate that during various growth stages of potatoes, all fertilization treatments (T1, T2, T3) significantly enhanced soil nitrogen transformation characteristics compared to the control group (CK) (P < 0.05). The total soil nitrogen (**Figure 1a**) content was higher in fertilized treatments than in CK. Notably, during the tuber expansion stage, the T3 treatment increased soil nitrogen content by 49.5% compared to CK and by 7.6% compared to T0. The largest difference in soil microbial biomass nitrogen (**Figure 1b**) was observed during the tuber formation stage, with T1 and T2 treatments showing significantly higher levels than others. T3 treatment excelled during the starch accumulation stage. During the tuber expansion stage, soil soluble organic nitrogen (**Figure 1c**) under T2 was significantly higher than under CK and T1, and remained higher than other treatments, except T3, during the starch accumulation stage. Soil nitrate (**Figure 1d**) and ammonium nitrogen (**Figure 1e**) contents generally decreased, but T2 and T3 treatments were slightly higher than others from the tuber formation stage onward. Overall, replacing 60% of chemical fertilizer nitrogen with organic fertilizer nitrogen (T2) significantly enhanced soil nitrogen transformation and accumulation capacity after the tuber formation stage. This suggests T2 has superior potential for nitrogen supply and maintenance, benefiting potato growth and nitrogen utilization.

**Figure 2:**
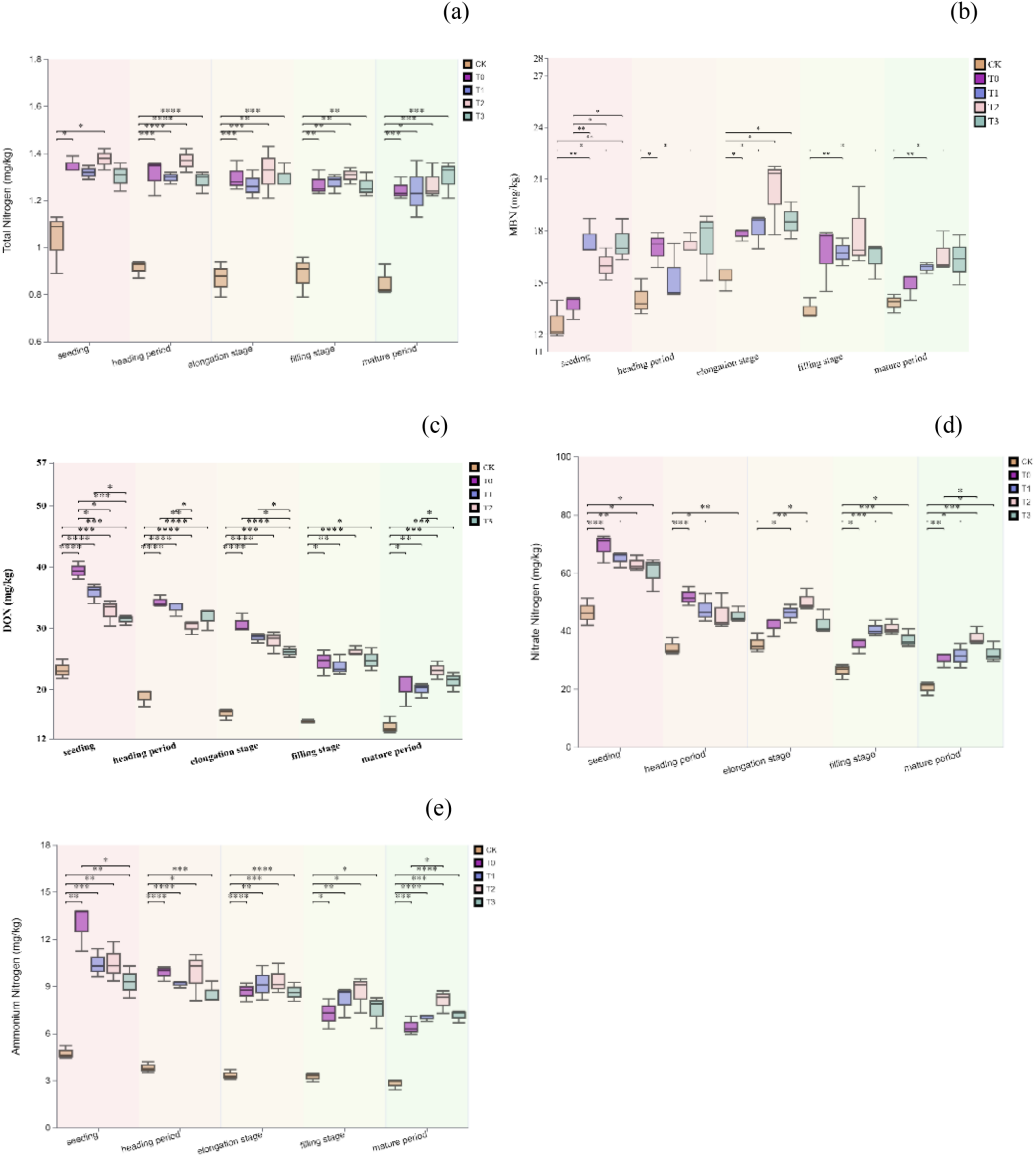
Total soil nitrogen **(a)**, Soil microbial biomass nitrogen **(b)**, Soil soluble organic nitrogen **(c)**, Soil nitrate nitrogen **(d)**, Soil ammonium nitrogen **(e)** during the wheat season. A single asterisk (*) denotes a significance level of p < 0.05; two asterisks (**) indicate p < 0.01; three asterisks (***) represent p < 0.005; and four asterisks (****) signify p < 0.001.

**Figure 2** illustrate significant variations in soil nitrogen transformation during the wheat season across different growth stages and fertilization treatments. Compared to the control (CK), all fertilization treatments notably increased the levels of soil total nitrogen, soil microbial biomass nitrogen, nitrate nitrogen, and ammonium nitrogen (P < 0.05). The T2 treatment demonstrated the highest nitrogen transformation efficiency at most growth stages. At the filling stage, total nitrogen and nitrate nitrogen levels in the T2 treatment were approximately 89.3% and 57.1% higher than those in the CK, respectively. Although ammonium nitrogen content remained relatively stable throughout the growth period, it was significantly higher in the T1, T2, and T3 treatments compared to the CK. Overall, the combined application of organic manure and chemical fertilizers (T2, T3) proved more effective for soil nitrogen transformation and accumulation than the sole use of chemical fertilizers (T0). This suggests that integrating organic and inorganic fertilizers positively impacts the soil’s nitrogen supply capacity.

### 3.3 Enzyme activities related to soil nitrogen transformation

**Figure 3:**
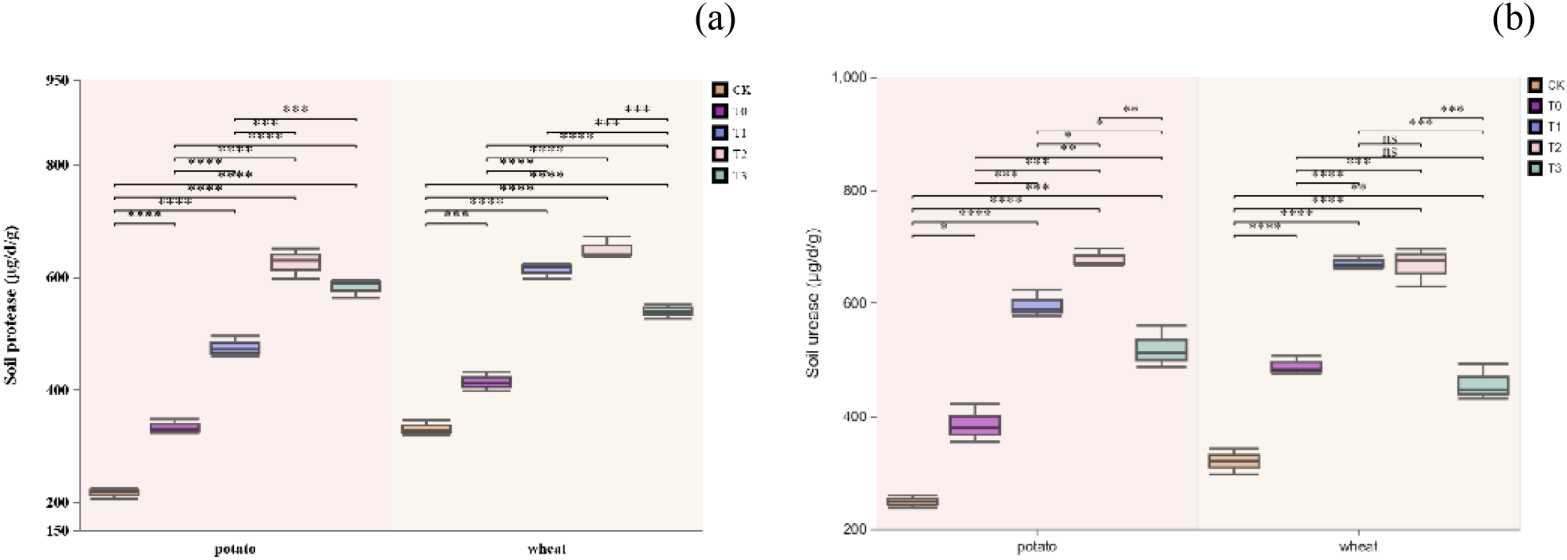
Soil protease **(a)** and Soil urease **(b)**. A single asterisk (*) denotes a significance level of p < 0.05; two asterisks (**) indicate p < 0.01; three asterisks (***) represent p < 0.005; and four asterisks (****) signify p < 0.001.

During the potato season, soil alkaline protease activity (ALPT) was notably higher in treatments T0 and T2 compared to other treatments. In contrast, during the wheat season, the activity followed the order: T3 > T2 > T0 > T1. For soil urease activity (SU) in the potato season, treatment T2 exhibited significantly higher activity than other treatments. In the wheat season, treatments T2 and T1 showed significantly higher activity than the others (**Figure 3**). These findings suggest that substituting an appropriate amount of organic fertilizer nitrogen for chemical fertilizer nitrogen can substantially enhance soil enzyme activity, thereby promoting organic matter decomposition and nitrogen release.

### 3.4 Nitrogen use efficiency

During both the potato and wheat growing seasons, treatment T2 achieved the highest nitrogen use efficiency (NUE), with values of 47.5% and 48.6%, respectively (**Table 6**). These figures were 73.4% and 76.1% higher than those of the full chemical fertilizer treatment (T0) (P < 0.05). In contrast, the NUE of treatment T3 significantly decreased, likely due to limited crop uptake resulting from insufficient nitrogen supply.

**Table 6:** The nitrogen use efficiency of different treatments.

Treatments	NUE (%)	
	Potato	Wheat
T0	27.4 ± 1.9 c	27.6 ± 0.7 c
T1	21.1 ± 1.4 c	35.8 ± 2.8 b
T2	47.5 ± 3.4 a	48.6 ± 5.5 a
T3	34.8 ± 4.4 b	29.4 ± 1.5 bc

### 3.5 Correlation between soil nitrogen content and soil enzyme activity

After Substituting Organic Fertilizer for Chemical Fertilizer Nitrogen In this section, we examine the relationship between soil nitrogen content and soil enzyme activity following the substitution of organic fertilizer for chemical fertilizer nitrogen. Our analysis aims to understand how this substitution influences soil health and nutrient cycling. Previous studies have established that organic fertilizers can enhance soil enzyme activity, which is crucial for nutrient mineralization and organic matter decomposition. In our experiments, we observed that replacing chemical fertilizers with organic alternatives significantly increased soil nitrogen levels. This increase, in turn, correlated with elevated enzyme activity, suggesting improved soil biological function. The data revealed that soils treated with organic fertilizers showed higher activities of enzymes such as urease and phosphatase compared to those treated with chemical fertilizers. These enzymes play vital roles in nitrogen and phosphorus cycling, respectively. The enhanced enzyme activity likely results from the increased availability of organic matter and nutrients provided by the organic fertilizers. Our findings indicate that substituting organic fertilizers for chemical nitrogen fertilizers can positively impact soil enzyme activity, thereby promoting better nutrient cycling and soil health. Future research should explore long-term effects and the potential for optimizing fertilizer combinations to maximize soil productivity and sustainability.

**Figure 4:**
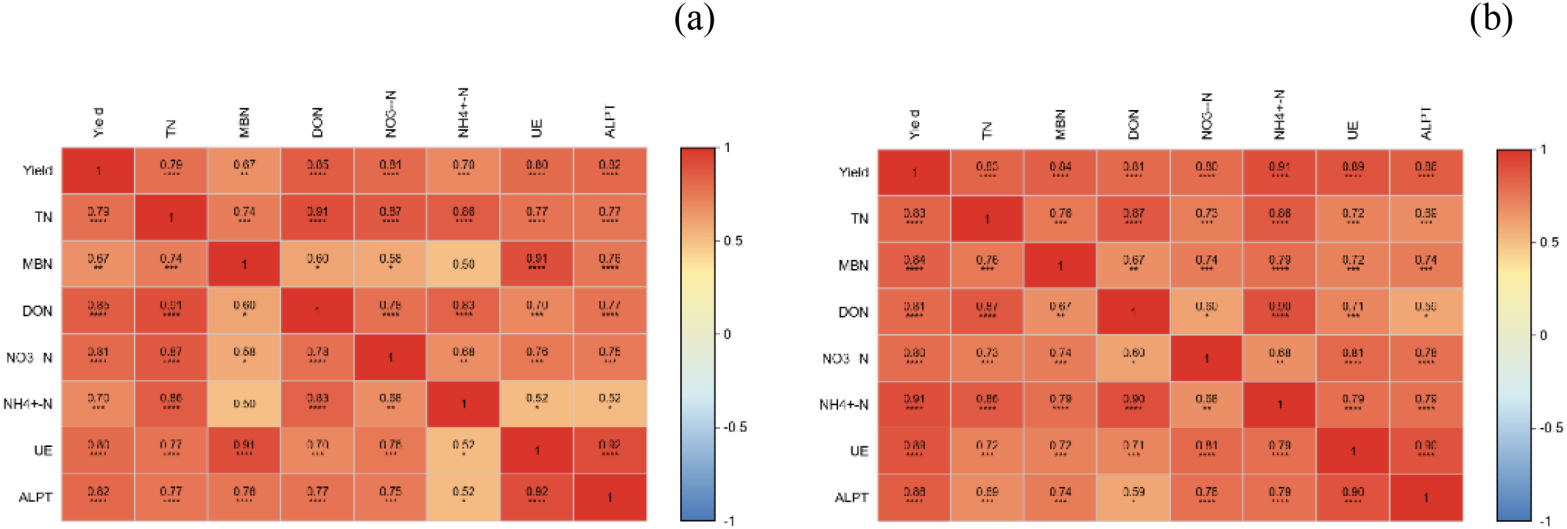
Correlation analysis between soil nitrogen forms and yield during the potato season and the wheat season.

**Figure 4**’s correlation analysis heat map illustrates the relationships among crop yields, soil enzyme activities, and nitrogen components. The findings reveal a significant positive correlation between the yields of potatoes (**Figure 4a**) and wheat (**Figure 4b**) with soil TN, NO_3_^-^-N, and NH_4_^+^-N (p < 0.01). Notably, the correlation between crop yields and soil urease surpasses that with soil sucrase. Specifically, wheat yield exhibits the highest correlation with soil urease (**Figure 4b**), with a correlation value of 0.82, highlighting soil urease activity as a potential key factor in wheat productivity. Additionally, the correlation between soil urease and soil nitrogen components is stronger than that between soil sucrase and nitrogen components. The study also found no correlation between soil urease and sucrase (r ≈ 0), indicating their functional independence in organic matter decomposition. 

**Figure 5:**
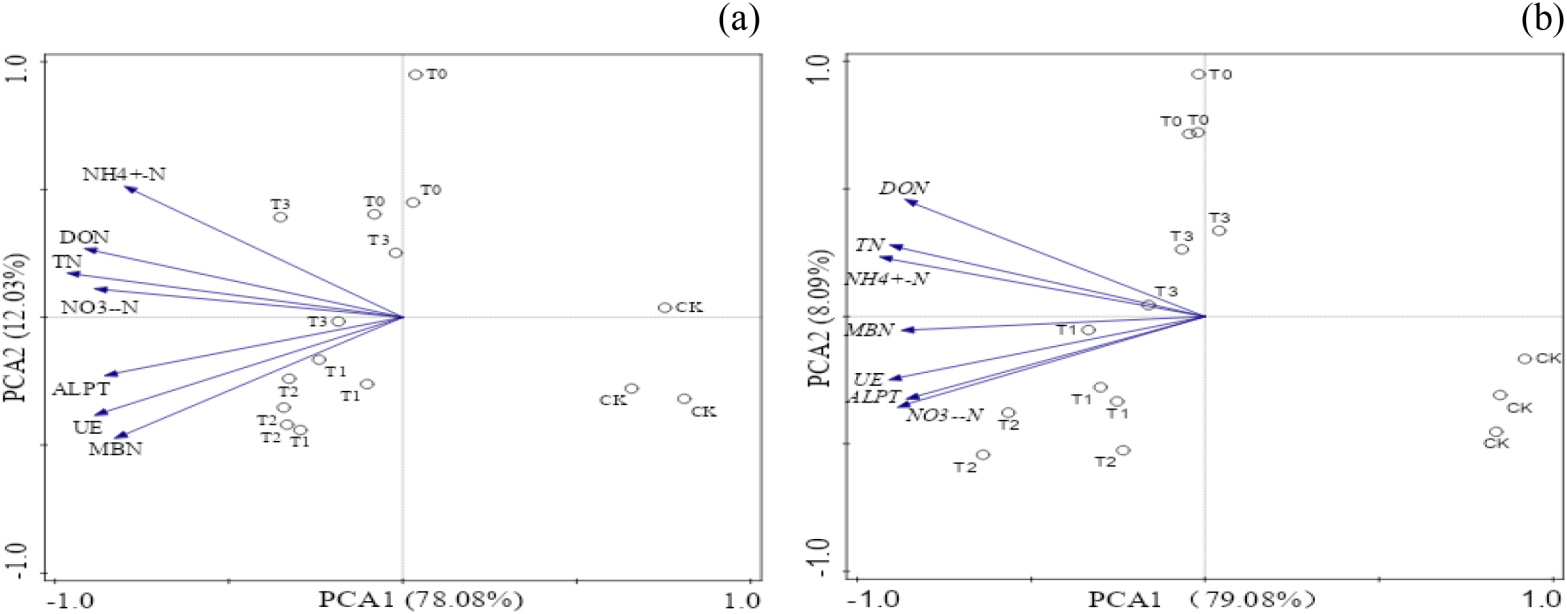
Presents a principal component analysis of various nitrogen forms and nitrogen hydrolases in soil throughout the **(a)** potato and **(b)** wheat growing season.

Principal component analysis revealed that PC1 and PC2 accounted for 78.08% and 12.03% of the variation in potato yield (**Figure 5a**), respectively. In the ordination plot, potato yields from the five treatments were distinctly separated. This indicates that partially replacing chemical fertilizer nitrogen with organic fertilizer nitrogen significantly affected potato yield. Principal component analysis revealed that PC1 and PC2 accounted for 79.08% and 8.09% of the variation in wheat yield (**Figure 5b**), respectively. The ordination diagram distinctly separated wheat yields across the five treatments, particularly between the CK and T0 treatments. This indicates that replacing chemical fertilizer nitrogen with organic manure nitrogen significantly impacted wheat yield. 

**Figure 6:**
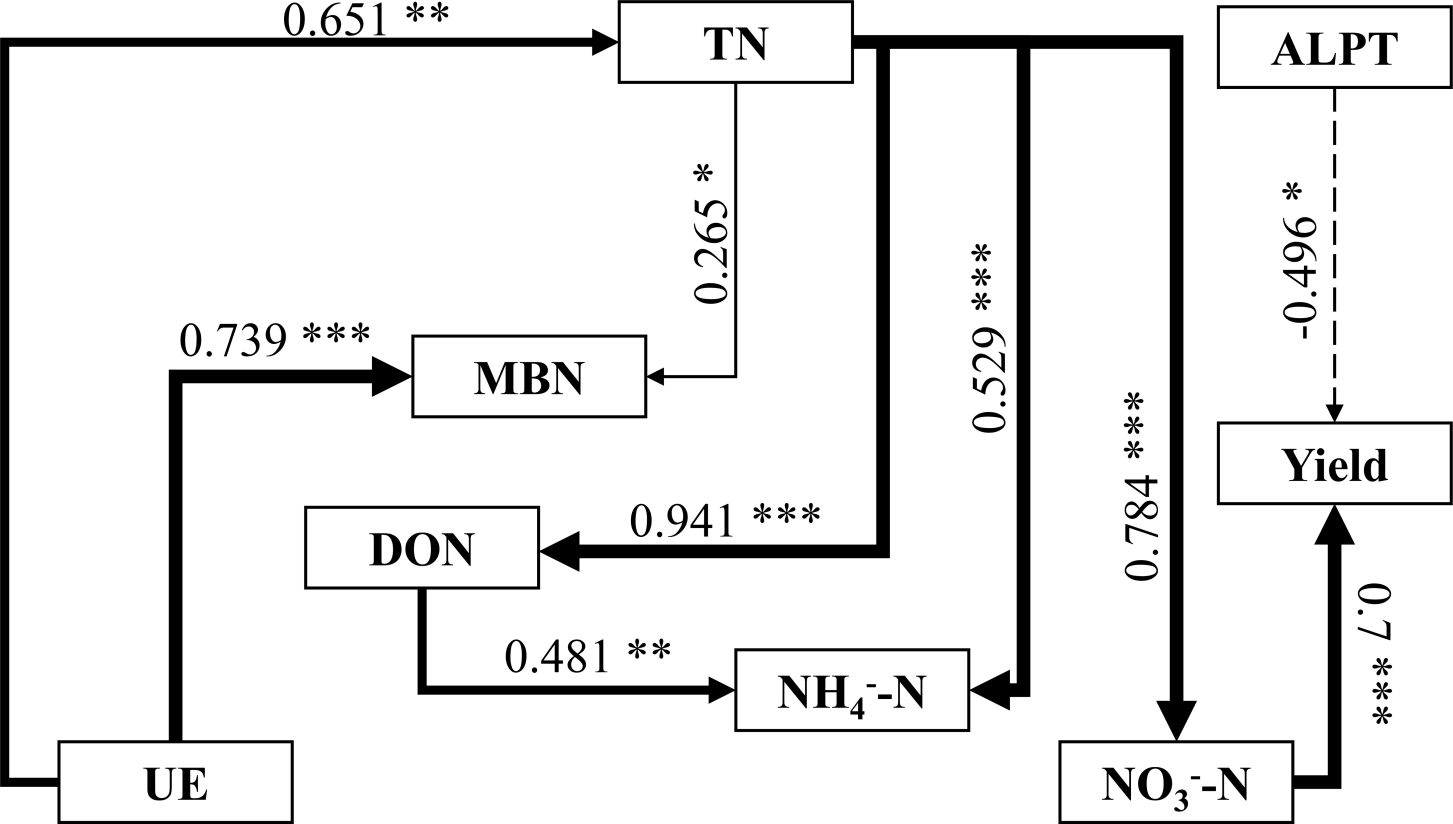
Illustrates the structural equation model linking soil nitrogen forms, nitrogen hydrolase activities, and crop yields. Note: The width of the arrows corresponds to the strength of the pathway coefficient. Solid arrows denote positive pathway coefficients, while dashed arrows indicate negative ones. Symbols *, **, and *** denote significance levels of P < 0.05, P < 0.01, and P < 0.001, respectively.

A structural equation model was employed to analyze the relationships among soil nitrogen hydrolase activities, soil nitrogen forms, and crop yield (**Figure 6**). Soil urease (UE) indirectly affected crop yield by first influencing soil total nitrogen, which subsequently impacted soil NO_3_^-^ - N. Conversely, soil protease exhibited a negative correlation with crop yield. These enzymatic interactions elucidate the quantitative association mechanism among soil enzyme activities, nitrogen forms, and crop yield.

## 4 Discussion

This study systematically evaluated the impact of substituting organic fertilizer for chemical nitrogen fertilizer on soil nitrogen transformation, enzyme activity, and crop yield within a potato-wheat rotation system. This evaluation was conducted through a two-year fixed-point field experiment. The discussion focuses on three main aspects: nitrogen supply characteristics, soil biological processes, and the synergistic mechanisms influencing crop responses. Furthermore, the research findings are analyzed in conjunction with regional ecological characteristics to highlight their scientific value and practical significance.

### 4.1 Alignment of nitrogen release from organic fertilizers with crop nitrogen demand

The study found that replacing 60% of chemical fertilizer nitrogen with organic fertilizer nitrogen significantly increased nitrate nitrogen content during the potato tuber expansion and wheat jointing stages, maintaining a relatively high mineral nitrogen level at maturity (**Figure 2**). This aligns with nitrogen release patterns from combined organic and inorganic fertilizers reported by ^(^Rongxiu et al., 2025^)^ and ^(^Hu et al., 2025^)^. An appropriate amount of chemical fertilizer provides readily available nitrogen early in crop growth, while organic fertilizer’s continuous mineralization sustains nitrogen supply in later stages. This approach better matches crop nutrient demands compared to the “excessive early, insufficient late” pattern of full chemical fertilizer use ^(^Tingcheng et al., 2024^;^Qiying et al., 2021^;^Zhenglin et al., 2024^)^. However, substituting 100% of chemical fertilizer nitrogen with organic fertilizer resulted in insufficient nitrogen during critical growth stages. Particularly in the second planting season, nitrate and ammonium nitrogen supplies decreased at multiple wheat growth stages. The study area, located at the northern foot of the Yinshan Mountains, experiences an arid, cool climate with low annual temperatures and precipitation, often resulting in low soil moisture. Previous research indicates that low temperatures and drought significantly inhibit soil microbial activity and organic matter mineralization, delaying nitrogen release from organic fertilizers and failing to meet early-stage crop growth needs ^(^Paul and Urs, 2021^;^Yibing et al., 2024^;^Yajun et al., 2024^)^. Therefore, in similar climates, relying solely on organic fertilizers may lead to early-stage nitrogen deficiencies. It is recommended to optimize the C/N ratio of organic fertilizers or introduce exogenous microbial agents to enhance mineralization efficiency.

### 4.2 Nitrogen transformation enhancement pathways driven by soil enzyme activities

Treatment T2, which replaced 60% of chemical fertilizer nitrogen with organic fertilizer, significantly increased the activities of soil urease (SU) and protease (ALPT) (**Figure 3**). Notably, SU activity showed a strong positive correlation with crop yield (r = 0.68–0.82). This finding aligns with previous reports indicating that organic fertilizer generally enhances soil enzyme activities. The introduction of organic fertilizer supplies an effective carbon source for microorganisms, potentially stimulating microbial growth, metabolic processes, and nitrogen transformation ^(^Xiaoxu et al., 2024^;^Bohan et al., 2021^)^. Interestingly, the enzyme activity response was more pronounced during the potato season compared to the wheat season. This difference may be attributed to the high nitrogen demand of tuber crops and the associated rhizosphere priming effect ^(^Gong et al., 2023^;^Kammoun et al., 2021^)^. Additionally, the weak correlation between SU and ALPT activities (r ≈ 0) suggests that these enzymes may independently govern nitrogen mineralization and carbon metabolism processes. Future research could employ methods like metagenomics to further explore the driving mechanisms of relevant functional microbial communities and enzyme activities.

### 4.3 Physiological and ecological basis for yield improvement

In terms of crop responses, the nitrogen use efficiency (NUE) of the T2 treatment increased by 73.4%–76.1% compared to the full chemical fertilizer treatment (**Table 6**). The yield-enhancing mechanisms include: (1) Optimization of nitrogen spatial distribution: Nitrogen accumulation in potato tubers increased by 8.5% (**Figure 1b**), and nitrogen distribution efficiency in wheat grains rose by 5.8% (**Figure 1d**), indicating improved nitrogen absorption by sink organs. (2) Improved synchronization of nitrogen supply with crop nitrogen demand: The slow-release properties of organic fertilizers aligned the peak nitrogen supply with the tuber expansion and grain filling periods, reaching an 82% overlap (**Figure 1**). This alignment alleviated issues of excessive early absorption and insufficient later supply typical of traditional chemical fertilizers. The initial soil organic matter content in the experimental area was relatively low at 2.02% (**Table 1**), significantly below the 4%–6% found in typical agricultural areas of the North China Plain. This low baseline may have amplified the positive effects of organic fertilizers on soil carbon-nitrogen coupling and crop responses, supporting the promotion of organic substitution strategies in nutrient-poor soils (Lu et al., 2023; Zeeshan Ul Haq et al., 2023).

### 4.4 Application insights for sustainable regional agricultural development

To fully grasp the long-term effects of substituting organic fertilizers on soil health and crop productivity, future research should examine soil carbon-nitrogen balance, microbial community succession, and nutrient cycling over extended periods. While this study highlights the immediate impacts of organic fertilizer substitution, the enduring effects on soil microbial diversity, enzyme activities, and ecosystem stability remain uncertain. Long-term experiments are essential to assess how organic fertilizers influence these dynamics and their relationship to soil carbon stock variations, offering deeper insights into sustainability and environmental degradation mitigation. Additionally, future research should explore how different organic fertilizers interact with varying climatic conditions, focusing on their potential to enhance soil carbon sequestration and improve resilience to climate change, especially in arid and semi-arid regions. Our findings suggest adopting a partial organic fertilizer substitution strategy, such as replacing approximately 60% of chemical nitrogen (N) with organic N, to reduce chemical N input while maintaining yield stability. This approach enhances soil nitrogen transformation capacity, boosts enzyme activities, and improves nitrogen use efficiency compared to full chemical fertilization. However, 100% substitution may risk insufficient early-season nitrogen supply under arid and cool conditions due to delayed mineralization. Future studies should further investigate the mineralization dynamics of various manure types and their interactions with local temperature and moisture conditions. Optimizing the combination of organic and chemical N is crucial to better synchronize soil nitrogen supply with crop nitrogen demand.

At the ecological level, the T2 treatment reduces chemical nitrogen fertilizer use by 60%, aligning with China’s strategy to “reduce use and increase efficiency of chemical fertilizers.” Organic fertilizer application increased ^(^Yang et al., 2023^)^ soil organic carbon content by an average of 12.9%. In regions such as the northern foot of the Yinshan Mountains, where farmland is prone to wind erosion and desertification, this practice has strong potential for soil conservation and carbon sequestration. Our study found that substituting chemical fertilizers with organic alternatives can enhance both crop yield and soil fertility. However, the long-term effects and the carbon-nitrogen balance threshold require ongoing observation. For future research, we propose the following: (1) Extend the experimental period to determine the stability and threshold effects of soil carbon pool accumulation; (2) Develop efficient microbial agents tailored for arid and cold regions to enhance organic fertilizer mineralization under challenging conditions; (3) Construct a dynamic prediction model for organic fertilizer replacement ratios. This model should integrate parameters such as basic soil fertility, precipitation patterns, and crop nitrogen requirements at various growth stages to optimize fertilization systems precisely.

## 5 Conclusions

This study demonstrates that in potato and wheat cultivation at the northern foot of the Yinshan Mountains in Inner Mongolia, substituting 60% of chemical fertilizer nitrogen with organic fertilizer nitrogen, while maintaining a consistent total nitrogen application rate, significantly boosts crop yield and nitrogen use efficiency. The observed increase in yield and efficiency results from the combined supply of fast-acting and slow-release nitrogen sources. This approach satisfies the peak nitrate nitrogen demand during potatoes’ tuber expansion and wheat’s jointing stages, while also maintaining a relatively high mineral nitrogen level (14% increase) in later growth stages. This fertilization strategy achieves a high yield level (47.13 t·ha^-^¹ for potatoes and 6.06 t·ha^-^¹ for wheat) by replacing 60% of chemical fertilizer nitrogen. It also shows potential for enhancing soil fertility, offering a viable cultivation strategy for reducing fertilizer use, increasing efficiency, and protecting farmland ecology in arid and cold regions. Future research should focus on developing functional microbial agents tailored to regional climate conditions to further enhance the nitrogen release efficiency of organic fertilizers.

## Data Availability

The original contributions presented in the study are included in the article/supplementary material. Further inquiries can be directed to the corresponding author.

## References

[B1] Ankita B. Mahadev P. Susanta D. Bappa P. Gopal D. Partha Sarathi P. . (2024). Inter-cropping patterns and nutrient management effects on maize growth, yield and quality. Field Crops Res. 310, 109363. doi: 10.1016/j.fcr.2024.109363, PMID: 41743167

[B2] Bohan W. He P. Mingping S. Huanyan L. Xitong W. Rong Z. . (2021). Evaluation of phytoremediation potential of native dominant plants and spatial distribution of heavy metals in abandoned mining area in Southwest China. Ecotoxicology Environ. Saf. 220, 112368. doi: 10.1016/j.ecoenv.2021.112368, PMID: 34082243

[B3] Chenxiao D. Jiabei L. Binbin Z. Shufang W. Junliang F. Hao F. . (2023). Effect of bio-organic fertilizer derived from agricultural waste resources on soil properties and winter wheat (Triticum aestivum L.) yield in semi-humid drought-prone regions. Agric. Water Manage. 289, 108539. doi: 10.1016/j.agwat.2023.108539, PMID: 41743167

[B4] Fengwei Y. Jianbin L. Yilu W. Zhongyi Y. (2024). Biodegradable chelating agents for enhancing phytoremediation: Mechanisms, market feasibility, and future studies. Ecotoxicology Environ. Saf. 272, 116113. doi: 10.1016/j.ecoenv.2024.116113, PMID: 38364761

[B5] Gholamreza G. Muhammad A. Muhammad Arslan A. Antonio J.-M. Andrzej K. (2024). Novel approaches to alleviate abiotic stresses in crop plants using new engineered nanoparticles. Plant Stress 13, 100554. doi: 10.1016/j.stress.2024.100554, PMID: 41743167

[B6] Gong X. Li S. Wu Z. Alhaj H. Y. Shaghaleh H. Kalkhajeh Y. . (2023). Biochar enhances soil resource availability and suppresses microbial metabolism genes in the rhizosphere of wheat. Life (Basel Switzerland) 13, 1843. doi: 10.3390/life13091843, PMID: 37763247 PMC10533193

[B7] Hu C. Sheng-Nan H. Xin-Yi W. Hui Z. (2025). Organic fertilizer-mediated cultivated land conservation and pollution source control in agricultural ecosystem, Northeast China. Environ. Technol. Innovation 37, 103945. doi: 10.1016/j.eti.2024.103945, PMID: 41743167

[B9] Jia W. Ma Q. Li L. Dai C. Zhu M. Li C. . (2023). The fate of nitrogen from different sources in a rice-wheat rotation system – A 15N labeling study. Front. Plant Sci. 14. doi: 10.3389/fpls.2023.1271325, PMID: 37929166 PMC10620804

[B8] Ju L. Xuemei X. Jian L. Chengfei G. Muhammad A. Guobin Z. . (2024). Integrating bio-organic fertilization increases twice-yearly cabbage crop production by modulating soil microbial community and biochemical properties in Northwest Plateau. Environ. Technol. Innovation 35, 103715. doi: 10.1016/j.eti.2024.103715, PMID: 41743167

[B11] Kammoun B. Journet E. Justes E. Bedoussac L. (2021). Cultivar grain yield in durum wheat-grain legume intercrops could be estimated from sole crop yields and interspecific interaction index. Front. Plant Sci. 12. doi: 10.3389/fpls.2021.733705, PMID: 34721461 PMC8551613

[B10] Kerner P. Struhs E. Mirkouei A. Aho K. Lohse K. Dungan R. . (2023). Microbial responses to biochar soil amendment and influential factors: A three-level meta-analysis. Environ. Sci. Technol. 57, 19838–19848. doi: 10.1021/acs.est.3c04201, PMID: 37943180 PMC10702529

[B12] Lu Z. Zhou Y. Li Y. Li C. Lu M. Sun X. . (2023). Effects of partial substitution of chemical fertilizer with organic manure on the activity of enzyme and soil bacterial communities in the mountain red soil. Front. Microbiol. 14. doi: 10.3389/fmicb.2023.1234904, PMID: 37736094 PMC10509364

[B13] Mateusz S. Derya Ç. Krzysztof T. Małgorzata M. Katarzyna C. (2024). Advancements in algal biorefineries for sustainable agriculture: Biofuels, high-value products, and environmental solutions. Biocatalysis Agric. Biotechnol. 58, 103224. doi: 10.1016/j.bcab.2024.103224, PMID: 41743167

[B15] Michail L. G. Paul J. B. Ruben S. Ann H. Helen S. Charles K. (2023). Modelling the effects of soil organic content and pH on the yield responses of tea to nitrogen fertilizer. Agric. Syst. 212, 103754. doi: 10.1016/j.agsy.2023.103754, PMID: 41743167

[B14] Muhammad Q. Dong-chu L. Jing H. Tian-fu H. Waqas A. Sehrish A. . (2022). Dynamics of organic carbon and nitrogen in deep soil profile and crop yields under long-term fertilization in wheat-maize cropping system. J. Integr. Agric. 21, 839–826. doi: 10.1016/s2095-3119(20)63501-8, PMID: 41647364

[B16] Paul H. Urs S. (2021). Simplifying residual nitrogen (Nmin); sampling strategies and crop response. Eur. J. Agron. 130, 126369. doi: 10.1016/j.eja.2021.126369, PMID: 41743167

[B17] Qinghua G. Wenliang W. (2024). Dynamics of soil water and nitrate within the vadose zone simulated by the WHCNS model calibrated based on deep learning. Agric. Water Manage. 292, 108653. doi: 10.1016/j.agwat.2023.108653, PMID: 41743167

[B18] Qiying Z. Hui Q. Panpan X. Kai H. Faxuan Y. (2021). Groundwater quality assessment using a new integrated-weight water quality index (IWQI) and driver analysis in the Jiaokou Irrigation District, China. Ecotoxicology Environ. Saf. 212, 111992. doi: 10.1016/j.ecoenv.2021.111992, PMID: 33529922

[B19] Rongxiu Y. Lulu L. Xin L. Huifang L. Jianmei Y. Chiyu M. . (2025). Positive effects of nitrogen fertilization on the flavor ingredients of tea (Wuniuzao), soil physicochemical properties, and microbial communities. Environ. Technol. Innovation 37, 103911. doi: 10.1016/j.eti.2024.103911, PMID: 41743167

[B21] Tingcheng Z. Aibin H. Mohammad Nauman K. Qi Y. Shaokun S. Lixiao N. (2024). Coupling of reduced inorganic fertilizer with plant-based organic fertilizer as a promising fertilizer management strategy for colored rice in tropical regions. J. Integr. Agric. 23, 107–193. doi: 10.1016/j.jia.2023.04.035, PMID: 41743167

[B20] Tufail M. Irfan M. Umar W. Wakeel A. Schmitz R. (2023). Mediation of gaseous emissions and improving plant productivity by DCD and DMPP nitrification inhibitors: Meta-analysis of last three decades. Environ. Sci. pollut. Res. Int. 30, 64719–64735. doi: 10.1007/s11356-023-26318-5, PMID: 36929253 PMC10172236

[B22] Ubaldi F. Frangella C. Volpini V. Fortugno P. Valeriani F. Romano S. V. (2023). Systematic review and meta-analysis of dietary interventions and microbiome in phenylketonuria. Int. J. Mol. Sci. 24, 17428. doi: 10.3390/ijms242417428, PMID: 38139256 PMC10744015

[B23] Xiaoxu Z. Jialiang Z. Shengjun X. Jinglin W. Faqian S. Yawen X. . (2024). Efficient partial denitrification-anammox process enabled by a novel denitrifier with truncated nitrite reduction pathway. Environ. Technol. Innovation 36, 103830. doi: 10.1016/j.eti.2024.103830, PMID: 41743167

[B25] Yajun Y. Hexiang L. Hui W. Chengjuan L. Jialong L. (2024). Strategies of soil microbial N-cycling in different cadmium contaminated soil with wheat straw return. Ecotoxicology Environ. Saf. 278, 116406. doi: 10.1016/j.ecoenv.2024.116406, PMID: 38728941

[B26] Yang Y. Ye C. Zhang W. Zhu X. Li H. Yang D. . (2023). Elucidating the impact of biochar with different carbon/nitrogen ratios on soil biochemical properties and rhizosphere bacterial communities of flue-cured tobacco plants. Front. Plant Sci. 14. doi: 10.3389/fpls.2023.1250669, PMID: 37790782 PMC10543665

[B24] Yibing L. Lixun Z. Jing W. Shan X. Zhengfang Z. Yuntao G. (2024). Activation of persulfate by a layered double oxide supported sulfidated nano zero-valent iron for efficient degradation of 2, 2′, 4, 4′-tetrabromodiphenyl ether in soil. Environ. Int. 194, 109098. doi: 10.1016/j.envint.2024.109098, PMID: 39579442

[B27] Zhenglin Z. Ignacio M. Bruce A. L. Bjoern Ole S. Cameron M. P. (2024). Opportunities for mitigating net system greenhouse gas emissions in Southeast Asian rice production: A systematic review. Agriculture Ecosyst. Environ. 361, 108812. doi: 10.1016/j.agee.2023.108812, PMID: 41743167

[B10000] Zeeshan Ul Haq M. Yu J. Yao G. Yang H. Iqbal H. A. Tahir H. (2023). A systematic review on the continuous cropping obstacles and control strategies in medicinal plants. Int J Mol Sci. 24(15), 12470. doi: 10.3390/ijms241512470, PMID: 37569843 PMC10419402

